# Relationship between quality of life in young adults and impulsivity/compulsivity^✰^

**DOI:** 10.1016/j.psychres.2018.11.059

**Published:** 2019-01

**Authors:** Samuel R. Chamberlain, Jon E. Grant

**Affiliations:** a Department of Psychiatry, University of Cambridge, and Cambridge and Peterborough NHS Foundation Trust, Cambridge, UK; b Department of Psychiatry & Behavioral Neuroscience, University of Chicago, Chicago, UL, USA

**Keywords:** Impulsive, Compulsive, Addiction, Functioning, Cognition, Padua, Barratt

## Abstract

Impulsive and compulsive symptoms often become apparent during young adulthood, which is a critical time for brain development and establishment of life goals. The aim of this study was to identify important associations with quality of life in young adults, across a range of clinical, questionnaire, and cognitive measures, focusing on impulsivity and compulsivity. Significant relationships between exploratory variables and quality of life were identified using Partial Least Squares (PLS). In the 479 participants (mean age 22.3 [SD 3.6] years), quality of life was best explained by a one-factor model (p < 0.001). Variables significantly associated with lower quality of life were: older age, greater alcohol consumption, and the presence of impulse control disorders (including gambling, compulsive buying, intermittent explosive disorder, compulsive sexual behavior, binge-eating, and skin picking), mood/anxiety disorders, post-traumatic stress disorder, and substance use disorder. Worse quality of life was also significantly explained by higher impulsiveness on the Barratt scale, and by relative impairments in extra-dimensional set-shifting and quality of decision-making. These findings suggest that impulse disorders merit more public health attention, especially problematic gambling. Performance on decision-making and set-shifting tasks also appears particularly important in understanding quality of life in young adults.

## Highlights

• This study examined measures associated with quality of life in young adults.
• Impulse control disorders were strongly associated with lower quality of life.
• Trait impulsiveness and cognition were also associated with worse quality of life.

## 1 Introduction

Young adulthood involves major changes in an individual's environmental milieu – it constitutes a time of transition during which people may become more independent from family (e.g. moving to university), begin substantive employment for the first time, and form life-long social relationships (including partnerships). As well as external changes, young adulthood is also a crucial time for brain development, both in terms of structure and function (Casey et al., 2017, Colver and Longwell, 2013, Sharda et al., 2015). Behavioral habits formed in youth often have longer-term implications and may persist over time, such as substance use disorders (Degenhardt et al., 2016), which in turn impact brain development and cognition (Cservenka and Brumback, 2017). Two key concepts of particular relevance to young adulthood and understanding forms of psychopathology are impulsivity and compulsivity. Impulsivity refers to behaviors (or tendencies towards behaviors) that are unduly hasty, risky, and that lead to negative longer-term outcomes (Evenden, 1999). Compulsivity refers to behaviors (or tendencies towards behaviors) that are rigid, repetitive, and functionally impairing (Robbins et al., 2012). In normative settings, adolescents and young adults are relatively impulsive but impulsivity may reduce over time (Mitchell and Potenza, 2014, Steinberg et al., 2009). Compulsivity is less well studied from a longitudinal point of view, though translational research posits that certain behaviors (notably substance use) may shift from being impulsive to being compulsive with time, as behaviors are repeated (Belin et al., 2008, Koob and Le Moal, 2008).

While the impact of mainstream mental disorders (mood, anxiety, and substance use disorders) on quality of life and functioning in young people has been extensively studied, other disorders especially the impulse control disorders have often been overlooked (Bell et al., 2013, Lipari and Hedden, 2013, Patel et al., 2016, Patel et al., 2007).

Therefore, the aim of this study was to explore clinical, personality, and cognitive measures associated with quality of life in young adults, with an emphasis on impulsivity and compulsivity. To achieve this end, we used the innovative statistical approach of partial least squares, which is useful when there are a relatively large number of variables compared to the sample size; and where data are likely to correlate and be non-normally distributed. We hypothesized that quality of life would be significantly associated with a range of disorders in young adults, but especially so for impulse control disorders, in addition to substance use, anxiety, and mood disorders. We further predicted that more severe impulsivity reflected by questionnaire and cognitive based measures would be associated with worse quality of life. By contrast, we predicted that compulsive symptoms would be relatively weakly associated with quality of life in this setting.

## 2 Methods

### 2.1 Participants

Young adults, aged 18–29 years, were recruited using media advertisements in a large US city. Advertisements asked subjects to participate in a research study exploring impulsive and compulsive behaviors. Inclusion criterion was gambling at least once in the past year (since the overall study was exploring gambling in young people). Subjects were excluded if they were unable to give informed consent, were unable to understand/undertake the study procedures, or were seeking treatment for any mental disorders. Prior to participation, written informed consent was provided. The study was approved by Institutional Review Board (University of Chicago). Participants were compensated with a $50 gift card for a local department store for taking part.

### 2.2 Assessments

Each participant attended the research laboratory on one occasion to complete questionnaires, a clinical interview, and neuropsychological testing. All procedures were conducted in a quiet environment. The following demographic data were collected: age, gender, number of times alcohol consumed per week on average, and education level. Quality of life was assessed using the Quality of Life Inventory (QOLI) (Frisch et al., 2005), which comprehensively measures overall life satisfaction and well-being, has excellent psychometric properties, and is sensitive to effects of disease on quality of life; and beneficial effects of treatments (Frisch et al., 2005).

Structured clinical interviews were conducted using the previously validated Mini International Neuropsychiatric Inventory (MINI) (Sheehan et al., 1998) and Minnesota Impulse Disorder Interview (MIDI) (Grant et al., 2005). The MINI identifies mainstream mental disorders, including mood and anxiety disorders, obsessive-compulsive disorder, eating disorders, antisocial personality disorder, and substance use disorders. The MIDI identifies impulse control disorders: compulsive buying, kleptomania, trichotillomania, intermittent explosive disorder, pyromania, gambling disorder, compulsive sexual behavior, binge-eating disorder, and skin picking disorder (Grant, 2008). Extent of gambling disorder symptoms were measured using the Structured Clinical Interview for Gambling Disorder (SCI-GD) (modified for DSM-5) (Grant et al., 2004), impulsivity was measured using the Barratt Impulsiveness Scale (BIS-11) (Barratt, 1965, Patton et al., 1995, Stanford et al., 2016), and obsessive-compulsive traits with the Padua inventory (Sanavio, 1988).

Neuropsychological testing focused on three domains, and was conducted using the Cambridge Neuropsychological Test Automated Battery (CANTABeclipse, version 3, Cambridge Cognition Ltd, UK): the Cambridge Gamble task (Rogers et al., 1999), Stop-Signal task (Aron et al., 2007), and Intra-Dimensional/Extra-Dimensional set-shift task (Owen et al., 1991). These cognitive domains were chosen given that they have often been implicated in the pathophysiology of impulsive, compulsive, and addictive disorders (Chamberlain et al., 2016, Goudriaan et al., 2005, Goudriaan et al., 2006, Goudriaan et al., 2014, Grant and Chamberlain, 2014, Grant et al., 2011, Potenza, 2007, Potenza, 2008).

On the Cambridge Gamble task, for each trial, ten boxes were shown, some blue and some red, with a token having been hidden behind one of these. The participant selected the color of box they believed a token was hidden behind, and then decided how many points to gamble on having made the correct decision. The main measures of decision-making on the task were the proportion of points gambled overall, the proportion of rational decisions made (proportion of trials when the volunteer opted for the color that was in the majority), and the extent of risk adjustment (the extent to which individuals modulated the amount gambled depending on the probability of making correct choices).

On the Stop-signal task, participants viewed a series of directional errors appearing one-per-time on the screen, and made speeded motor responses – if a left arrow occurred, they pressed a left button, and vice versa for right facing arrows. When an auditory stop-signal (“beep” ) occurred, participants attempted to withhold their motor response for the given trial. The main outcome measure on the task is the stop-signal reaction time, which is an estimate of how long it takes a given individual to suppress an already triggered response.

On the Intra-Dimensional/Extra-Dimensional set-shift task, volunteers attempted to learn an underlying rule about which of two stimuli presented on the computer screen was correct. After making each choice by touching the stimulus, feedback was given (‘correct’ or ‘incorrect’ appeared on the screen). Through trial and error, participants learnt the underlying rule. Over the course of the task, the rule was changed by the computer to assess different components of flexible responding. The crucial task stage is the extra-dimensional shift stage, in which volunteers must shift attentional focus away from a previously relevant stimulus dimension onto a previously irrelevant stimulus dimension (the ‘extra-dimensional’ attentional shift). The key outcome measure on the task was the number of errors made on this stage.

### 2.3 Data analysis

To identify demographic, clinical, and cognitive measures associated with statistical variation in quality of life, we utilized the statistical technique of partial least squares (PLS) (Abdi and Williams, 2013, Cox and Gaudard, 2013, Garthwaite, 1994, Höskuldsson, 1988). This powerful statistical technique constructs one or more latent variables (known as PLS components) that optimally explain the relationship between a set of X variables (explanatory variables) and one of more Y variables (outcome variables). Here, the Y variable was quality of life, and the X variables were: age, gender, education level, number of times alcohol consumed per week, presence (or not) of each mental disorder identifiable by the MINI and MIDI, total gambling disorder symptoms endorsed (SCI-GD), Barratt impulsivity (motor, attentional, and planning), obsessive-compulsive traits (Padua total score), and the cognitive outcome measures for response inhibition, decision-making, and extra-dimensional set-shifting. PLS is ideal in situations in which variables are correlated with each other; and when the number of variables is large in comparison to the number of cases.

Analysis was conducted using JMP Pro software Version 13.0 (SAS Institute Inc., 2017). Any missing data points were imputed automatically by JMP using study means. The PLS model was fitted using leave-one-out cross-validation (non-linear iterative partial least squares, NIPALS algorithm), and the optimal number of latent factors was selected by minimizing the predictive residual sum of the squares (PRESS). Initial explanatory variables that did not pass the Variable Importance Threshold (VIP) of 0.8 were not retained in the model (2017). Explanatory variables significantly contributing to the model (i.e. explaining significant variance in quality of life) were identified on the basis of 95% confidence intervals for bootstrap distribution of the standardized model coefficients not crossing zero (N = 1000 bootstraps).

## 3 Results

The total sample size was 479 individals, with mean (standard deviation, SD) age 22.3 (3.6) years, 167 (33.8%) female. The average level of education was mean 3.2 (0.8), equivalent to high school or better. The number [percentage] of individuals in a given quality of life category based on norms was: high 56 [11.7%], normal 264 [55.1%], low 65 [13.6%], and very low 94 [19.6%]. The other characteristics of the sample are displayed in Table 1.

**Table 1:** Characteristics of the sample.

Measure	Mean (SD) or N [%]	Indicative normative data (where available)	Reference for normative data
Alcohol consumption, times per week	1.40 (1.40)	Highly variable across studies	
Presence of mainstream mental disorder (MINI)	173 [35.1%]	27.8% ∼	(Gustavson et al., 2018)
Presence of impulse control disorder (MIDI)	55 [11.4%]	10.4%	(Odlaug and Grant, 2010)
SCI-GD, symptoms endorsed	1.1 (2.0)	0.14 (0.8)	Unpublished (independent) young adult cohort
Barratt motor impulsivity	23.8 (4.7)	21.5 (4.0)	(Reise et al., 2013)
Barratt attentional impulsivity	16.9 (4.1)	14.4 (3.5)	(Reise et al., 2013)
Barratt non-planning impulsivity	23.7 (5.3)	23.3 (4.6)	(Reise et al., 2013)
Padua OC total score	19.6 (44.2)	46.8 (26.2)	(Sanavio, 1988)
SST Stop-signal inhibition, msec	181.5 (65.0)	167.8 (48.6)	(Chamberlain et al., 2006)
CGT, points gambled (%)	91.0 (1.3)	65 (1.3)	(Mannie et al., 2015)
CGT, rational decision-making (%)	95.0 (0.1)	99.0 (0.4)	(Mannie et al., 2015)
CGT, risk adjustment	1.53 (1.18)	1.8 (0.1)	(Mannie et al., 2015)
IED ED errors	9.7 (10.2)	10.3 (13.1) #	(Chamberlain et al., 2006)

Table Footer: Abbreviations: MINI = Mini International Neuropsychiatric Inventory; MIDI = Minnesota Impulse Disorders Inventory; SCI-GD = Structured Clinical Interview for Gambling Disorder; OC = Obsessive-compulsive; SST = Stop-Signal Task; CGT = Cambridge Gamble Task; IED = Intra-Dimensional/Extra-Dimensional Shift Task; ED = Extra-dimensional set-shift. # Errors to criterion calculated from trials to criterion. ∼ Prevalence estimate for any mental disorder (anxiety, mood, or SUDs).

**Fig. 1:**
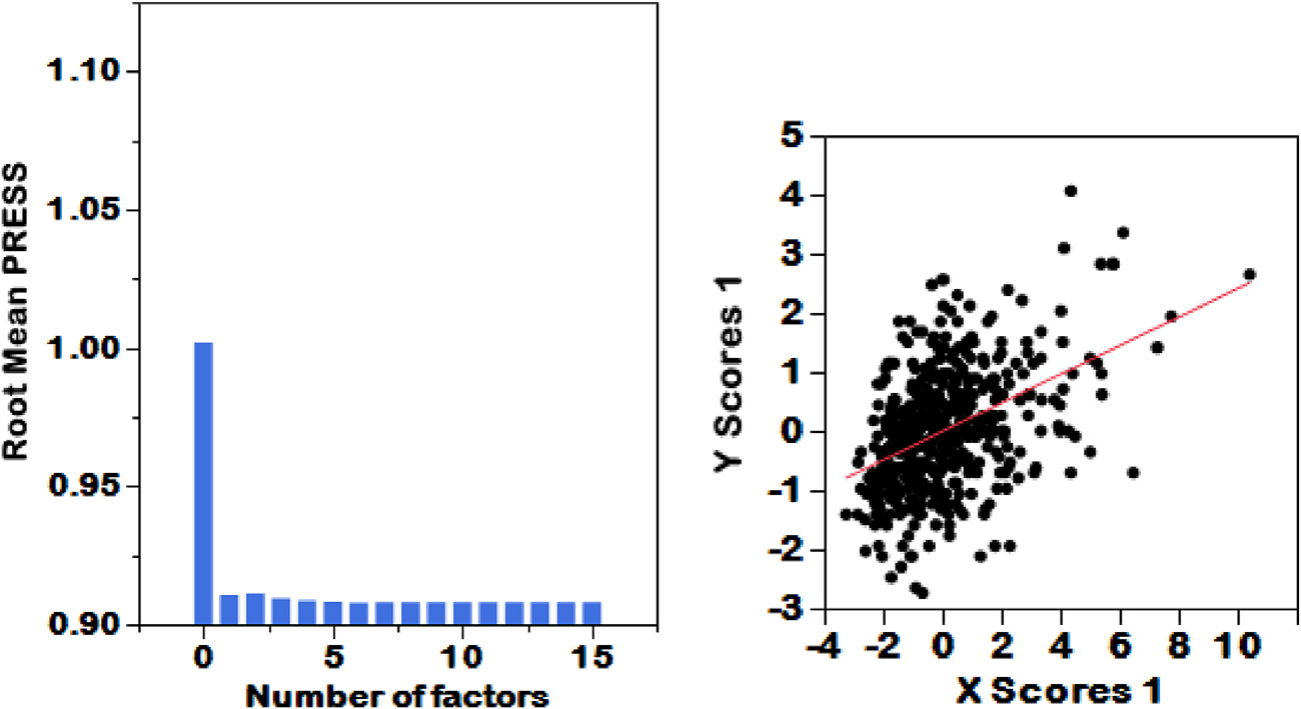
Left: Partial residual sum of the squares (PRESS) plot, showing that the optimal number of factors was one. Right: plot of explanatory factor scores (*X*) against quality of life factor score (*Y*) indicating good fit.

**Fig. 2:**
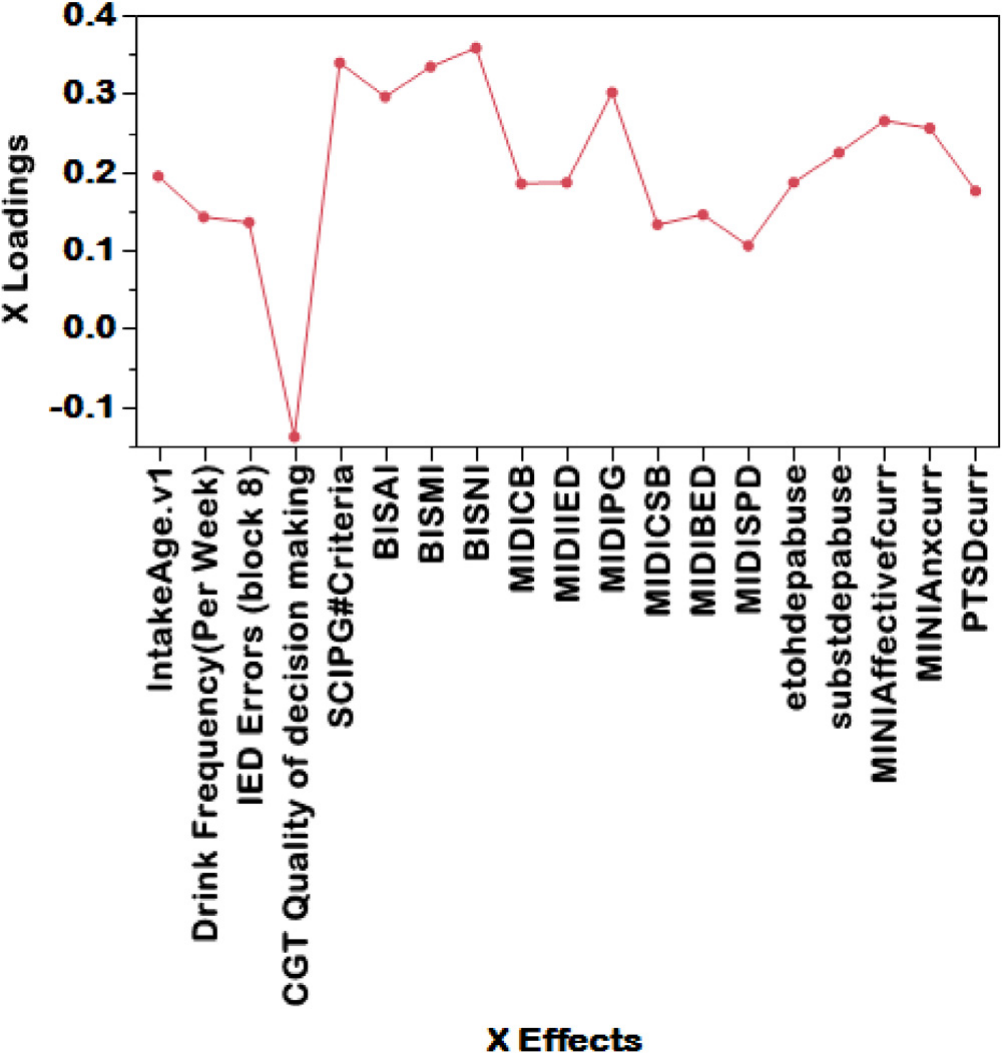
Results of the PLS model. The *X* axis lists demographic, clinical, and cognitive measures that were significantly statistically predictive of quality of life. The *y*-axis shows loadings of each variable onto the model (+ ve loadings indicate worse quality of life; -ve loadings better quality of life). IED: Intra-Dimensional/Extra-Dimensional set-shift task (extra-dimensional errors); CGT: Cambridge Gamble Task; SCIPG: maladaptive gambling scores on the Structured Clinical Interview for Gambling Disorder; BISAI: Barratt attentional impulsiveness; BISMI: Barratt motor impulsiveness; BISNI: Barratt non-planning impulsiveness; MIDICB: Minnesota Impulse Disorder Interview compulsive buying disorder; MIDIED: MIDI intermittent explosive disorder; MIDIBED: MIDI binge-eating disorder; etohdepabuse: alcohol use disorder on the Mini International Neuropsychiatric Inventory; substdepabuse: substance use disorder (besides alcohol) on the MINI; MINIAffectivecurr: mood disorder; MINIAnxcurr: anxiety disorder; PTSDcurr: post-traumatic stress disorder.

Partial Least Squares (PLS) yielded an optimal one-factor model (Fig. 1), which explained 17.8% of variance in the explanatory variables, and 19.7% of variance in quality of life. Inspection of residual and quantiles plots showed good fit and not significant outliers. Explanatory demographic, clinical, and cognitive measures that were significant in the PLS model are shown in Fig. 2.

For demographic measures, worse quality of life was associated with older age, and higher alcohol consumption per week. For clinical measures, worse quality of life was associated with presence of impulse control disorders (specifically gambling disorder, compulsive buying disorder, intermittent explosive disorder, compulsive sexual behavior disorder, skin picking disorder, and binge-eating disorder), substance use disorder (alcohol or other), any mood disorder, any alcohol disorder, and post-traumatic stress disorder. The relationship with gambling disorder was also significant on the dimensional SCI-GD measure of disordered gambling symptomatology. For questionnaires, higher scores on the Barratt impulsiveness scale were associated with lower quality of life. For cognitive functioning, extra-dimensional set-shifting impairment, and irrational decision-making (Cambridge Gamble Test), were both significantly associated with lower quality of life. Other X measures of interest were not significant contributors to the PLS model.

## 4 Discussion

Early adulthood is a crucial period, when young people may be exposed for the first time to a degree of independence and outlets for impulsive and compulsive behaviors (such as availability of psychoactive substances or gambling opportunities). This study explored ways in which quality of life was associated with a variety of such measures in young adults. We used the technique of partial least squares, which fits an optimal model best explaining variation in quality of life, based on explanatory variables, usefully accounting for inter-relationships between variables. The main finding was that worse quality of life was most strongly and significantly associated with disordered gambling symptoms, impulsive personality traits on the Barratt impulsivity scale, followed by mood, anxiety, and substance use disorders. Also significantly associated with worse quality of life were presence of certain impulse control disorders (compulsive sexual behavior disorder, binge-eating disorder, skin-picking disorder, compulsive buying disorder, and intermittent explosive disorder) along with worse extra-dimensional set-shifting, and older age.

That mood, anxiety, and substance use disorders were significantly and relatively strongly associated with worse quality of life in young adults was as expected. The public health impact of these disorders is widely recognized (Baxter et al., 2014, Patel et al., 2016). Our findings extend beyond these more traditionally recognized mental health disorders into the domain of impulsive and behaviorally addictive disorders, which are frequently overlooked both from a clinical point of view but also in terms of research funding. Problematic gambling is a major public health concern. In a systematic review of the literature, prevalence of problem gambling was estimated to be 3.1% globally (Ferguson et al., 2011). Meta-analysis focusing on studies conducted in college students found particularly high prevalence rates, of 6% for gambling disorder and 10% for problematic gambling (Nowak, 2017). Here, any level of disordered gambling (based on the total number of DSM criteria for gambling disorder endorsed) was associated with worse quality of life, as was a diagnosis of gambling disorder itself. This suggests that even milder forms of disordered gambling may have incremental negative effects on quality of life for young adults – higher even than other mental disorders that are more widely screened for in clinical practice such as mood and anxiety disorders. Gambling symptoms (number of criteria endorsed) had one of the strongest associations with quality of life compared to other variables examined, ranking similarly highly as impulsive personality traits measured using the Barratt impulsivity scale.

DSM diagnoses of intermittent explosive disorder, binge-eating disorder, and skin picking disorder were all linked with worse quality of life. Prior data are consistent with this finding. Binge-eating disorder is in fact the most common eating disorder globally (Kornstein, 2017). The majority of people with binge-eating disorder experience functional impairment especially in the domain of social functioning but also, to a lesser degree, in home and work settings (Kornstein, 2017). As well as the psychological impact, binge-eating disorder can lead to obesity, diabetes, and sleep disruption, which may feed into these quality of life associations. Quality of life impairment was previously compared in skin picking disorder, trichotillomania, and healthy controls. Both clinical groups had impaired quality of life but there was more psychosocial impact in skin picking disorder (Odlaug et al., 2010). This may partly account for why trichotillomania was not significantly associated with lower quality of life in our analysis; but another explanation is that trichotillomania was uncommon in our sample. In a recent review, the authors noted that there has been little scientific scrutiny of intermittent explosive disorder, with most published data being from one research site . In one of the first studies to examine intermittent explosive disorder, the majority of affected individuals reported significant distress, social impairment, vocational impairment, and legal consequences (McElroy et al., 1998). In view of recent advances in refinement of diagnostic criteria and neuroscientific research (Coccaro, 2012), the current study highlights the need for greater awareness of this condition as, in our experience, few mental health clinicians know about the disorder let alone screen for it.

Several other impulse control disorders were also associated here with lower quality of life: compulsive sexual behavior disorder and compulsive buying disorder. These conditions are not yet recognized explicitly in the DSM, but merit further consideration for inclusion in diagnostic classification systems based on the current results and previous findings (Black, 2001, Derbyshire and Grant, 2015). When individuals with compulsive buying disorder were followed up over five years, their symptoms had improved but had not abated – i.e. they were likely to still be functionally impaired (Black et al., 2016). Interestingly, in a large treatment-seeking sample of people with compulsive buying disorder, particularly high comorbidity was observed with compulsive sexual behavior, and intermittent explosive disorder (Nicoli de Mattos et al., 2016).

Measurement of impulsivity can be conducted not only at the level of overt psychiatric symptoms but also from the perspective of underlying intermediate phenotypes, such as questionnaires and neurocognitive testing (Grant and Chamberlain, 2014, Stanford et al., 2016). By examining brain-relevant processes cutting across mental disorders, it has been argued that psychiatry will make new inroads into understanding mental disorders and treating them (Insel et al., 2010). Of all the measures examined, Barratt impulsiveness scale scores loaded very highly onto the latent factor responsible for variation in quality of life in the partial least squares model; indeed, non-planning impulsiveness on this scale was the largest single determinant of lower quality of life in this sample. Barratt impulsiveness is useful as a candidate intermediate marker in psychiatry because it appears to be significantly heritable (Niv et al., 2012) and also has been linked with a number of genes (Gray et al., 2017, MacKillop et al., 2016).

Some of the cognitive measures were also associated with lower quality of life, to a significant but lesser degree, specifically worse quality of decision-making on the Cambridge Gamble Task, and more extra-dimensional set-shifting errors on the Intra-Dimensional/Extra-Dimensional set shift task. These tasks are dependent on the integrity of the medial and lateral prefrontal cortices respectively (Clark et al., 2004, Hampshire and Owen, 2006). Overall, the findings are in keeping with some people being predisposed towards impulsivity, which may reflect dysfunction of frontal brain regions, such as due to changes in developmental pathways. Contrary to expectation however, we did not find a significant relationship between quality of life and response inhibition measured by the Stop-signal test, which is a widely used measure of inhibition of pre-potent motor responses; nor between quality of life and obsessive-compulsive traits as indexed by the Padua inventory. Of note, the Padua inventory is designed to capture obsessive-compulsive symptoms rather than the broader concept of compulsivity. In future, scales designed to more fully capture compulsivity may enable closer inspection of the effects of such compulsive tendencies on quality of life.

Several limitations should be considered. The statistical model accounted for 17.8% of variance in the explanatory measures, and 19.7% of variance in quality of life. We feel this is likely to be clinically relevant, but this does mean that the majority of variance was theoretically explained by factors not assessed in this study. This is not surprising, given that quality of life is likely to be associated with a swathe of social, cultural, economic, mental health, and physical health factors. For questionnaires and cognitive testing, we focused on measures relevant to impulsivity, compulsivity, and addiction; as such, the scope of the project was restricted. This was not a comprehensive assessment of all mental health issues that may impact quality of life. The technique of PLS has advantages over more traditional statistical approaches (viz regression) in its ability to robustly handle correlations across explanatory variables and where there are relatively large numbers of explanatory variables; however, PLS may overlook more subtle correlations (Cramer, 1993). The study cannot address causality because it was cross-sectional rather than longitudinal in nature. Future work could study quality of life and its relationship with explanatory variables over time, to clarify cause and effect. The sample size may limit power. As can be seen in Table 1, the current sample for the most part had relatively normal scores / rates of endorsement compared to control data elsewhere. The exceptions to this were that the sample had relatively lower OC symptoms and relatively higher gambling of points (Cambridge Gamble Task) and higher endorsement of Gambling Disorder symptoms than would be anticipated based on other normal data. We suspect this is due to the recruitment method, which focused on young adults who gamble at least 5 times per year. This may limit generalizability of the results to the population at large. Lastly, we did not measure duration of different illnesses, and chronicity has been associated with cumulative untoward effects on quality of life.

In summary, this study highlights that certain facets of impulsivity (especially impulsive personality tendencies, and symptoms of disordered gambling and some impulse control disorders) bear strong associations with lower quality of life in young adults. These relationships appear more marked even than for mood, anxiety, and substance use disorders. Given that impulsive problems are often overlooked in clinical practice, the data highlight the importance of screening for such problems and intervening with a view to maximizing quality of life. Clinical trials should also consider incorporating measures such as the Barratt scale and scales measuring compulsivity once they are in future developed. It would be interesting to consider in future work whether impulsivity exerts a disproportionate burden on quality of life in distinct age groups; and indeed whether impulsivity in young people is associated with worse quality of life in later adulthood, even if impulsivity has lessened with time.
